# Supplementary material for: High-resolution transport-of-intensity quantitative phase microscopy with annular illumination
Source: Sci Rep. 2017 Aug 9;7:7654. doi: 10.1038/s41598-017-06837-1 (PMC5550517; doi:10.1038/s41598-017-06837-1)
Supplement: Supplementary file 1 — Supplementary Information [file 41598_2017_6837_MOESM1_ESM.pdf]

Supplementary Information:

High-resolution transport-of-intensity quantitative phase microscopy with annular illumination

Chao Zuo[1,2,*], Jiasong Sun[1,2], Jiaji Li[1,2], Jialin Zhang[1,2], Anand Asundi[3], and Qian Chen[2,*]

[1]Smart Computational Imaging (SCI) Laboratory, Nanjing University of Science and Technology, Nanjing, Jiangsu Province 210094, China
[2]Jiangsu Key Laboratory of Spectral Imaging & Intelligent Sense, Nanjing University of Science and Technology, Nanjing, Jiangsu Province 210094, China
[3]Centre for Optical and Laser Engineering (COLE), School of Mechanical and Aerospace Engineering, Nanyang Technological University, Singapore 639798, Singapore
[*]Correspondence and requests for materials should be addressed to C.Z. (email: zuochao@njust.edu.cn) or Q.C. (email: chenqian@njust.edu.cn)

ABSTRACT

This document provides supplementary information for "High-resolution transport-of-intensity quantitative phase microscopy with annular illumination".

Contents:

A. Derivation of Image Formation in a Partially Coherent Microscope
B. In-focus WOTF of a Partially Coherent Microscope
C. Comparison between Annular illumination TIE with Circular Illumination TIE in the Presence of Systematic Errors
D. Simulation of Phase Reconstruction for Non-weak Phase Object
E. Supplementary Videos

A. Derivation of Image Formation in a Partially Coherent Microscope

In the 6f optical configuration described in Figure 1, a thin complex object $t(\mathbf{x})$ is illuminated by the light emerging from an incoherent source with intensity distribution $S(\mathbf{u})$. Based on the van Cittert-Zernike theorem[1,2], the mutual intensity of the illumination just before the object is given by the Fourier transform of the source intensity distribution:

$$J_S(\mathbf{x}_1,\mathbf{x}_2) = J_S(|\mathbf{x}_1-\mathbf{x}_2|) = \iint S(\mathbf{u})e^{i2\pi\mathbf{u}(\mathbf{x}_1-\mathbf{x}_2)}d\mathbf{u} \tag{S1}$$

Then, the mutual intensity just behind the object is given by:

$$J_O(\mathbf{x}_1,\mathbf{x}_2) = J_S(\mathbf{x}_1,\mathbf{x}_2)\,t(\mathbf{x}_1)\,t^*(\mathbf{x}_2) \tag{S2}$$

The mutual intensity now depends on the object transmission [$t(\mathbf{x}_1)$ and $t^*(\mathbf{x}_2)$] and the mutual intensity of the illumination. After Fourier transform, the mutual intensity in the objective pupil ($\tilde{J}_O(\mathbf{u}_1,\mathbf{u}_2)$) is multiplied twice by the coherent transfer function $P(\mathbf{u})$, and then followed by an inverse transform to finally obtain for the mutual intensity in the image plane

$$J_I(\mathbf{x}_1,\mathbf{x}_2) = \iint \tilde{J}_O(\mathbf{u}_1,\mathbf{u}_2)\,P(\mathbf{u}_1)\,P^*(\mathbf{u}_2)e^{i2\pi(\mathbf{u}_1\mathbf{x}_1+\mathbf{u}_2\mathbf{x}_2)}d\mathbf{u}_1d\mathbf{u}_2 \tag{S3}$$

Equation S3 suggests that for partially coherent imaging, the mutual intensity $\tilde{J}_O(\mathbf{u}_1,\mathbf{u}_2)$ is transferred linearly with the mutual coherent transfer function, defined as $P(\mathbf{u}_1)\,P^*(\mathbf{u}_2)$. It can also be written as a two-fold convolution of the object mutual intensity $J_O(\mathbf{x}_1,\mathbf{x}_2)$ with the mutual point spread function, defined as $h(\mathbf{x}_1)\,h^*(\mathbf{x}_2)$[3]:

$$J_I(\mathbf{x}_1,\mathbf{x}_2) = \iint J_O(\mathbf{x}'_1,\mathbf{x}'_2)\,h(\mathbf{x}_1-\mathbf{x}'_1)\,h^*(\mathbf{x}_2-\mathbf{x}'_2)d\mathbf{x}'_1d\mathbf{x}'_2 \tag{S4}$$

This suggests that the mutual intensity in object space $J_O(\mathbf{x}_1,\mathbf{x}_2)$ is blurred by the amplitude point spread function in both coordinates ($\mathbf{x}_1$ and $\mathbf{x}_2$). The image intensity $I(\mathbf{x})$ is given by the values "on the diagonal" of the mutual intensity with

$$I(\mathbf{x}) = J_I(\mathbf{x},\mathbf{x}) = \iint J_S(\mathbf{x}_1,\mathbf{x}_2)\,t(\mathbf{x}_1)\,t^*(\mathbf{x}_2)\,h(\mathbf{x}-\mathbf{x}_1)\,h^*(\mathbf{x}-\mathbf{x}_2)d\mathbf{x}_1d\mathbf{x}_2 \tag{S5}$$

Substituting Equation S1 into Equation S5, the expression for the intensity can be simplified consequently, and Equation 1 is then derived

$$I(\mathbf{x}) = \int S(\mathbf{u})\left|\int t(\mathbf{x}')\,h(\mathbf{x}-\mathbf{x}')\,e^{i2\pi\mathbf{u}\mathbf{x}'}d\mathbf{x}'\right|^2 d\mathbf{u} \equiv \int S(\mathbf{u})I_{\mathbf{u}}(\mathbf{x})\,d\mathbf{u} \tag{S6}$$

B. In-Focus WOTF of a Partially Coherent Microscope

This section describes the in-focus WOTF of a partially coherent microscope, which is significantly dependent on the form and size of the light source (coherent parameter). When coherence parameter $s \geqslant 1$, the system behaves the same as an incoherent microscope with the WOTF given by the autocorrelation of the pupil function (Equation 16). For a prefect in-focus system (no pupil aberrations), the WOTF becomes a convolution of two circles of equal size[4], which can be represented as:

$$WOTF_{\text{incoh}}(\bar{\mathbf{u}}) = \frac{2}{\pi}\left[\arccos\left(\frac{\bar{\mathbf{u}}}{2}\right) - \frac{\bar{\mathbf{u}}}{2}\sqrt{1-\left(\frac{\bar{\mathbf{u}}}{2}\right)^2}\right] \qquad |\bar{\mathbf{u}}| < 2 \tag{S7}$$

where $\bar{\mathbf{u}}$ is the normalized spatial frequency with respect to the coherent resolution limit (NA_{obj}/λ). Figure S1(a) gives the in-focus $WOTF_{\text{incoh}}$, which is purely real-valued and can only image the absorption part. It can be seen that the absorption contrast gradually reduces as the increase in spatial frequency, with a 61% relative contrast loss at the coherent resolution limit (NA_{obj}/λ), and finally diminishes to zero in the incoherent resolution limit at ($2NA_{obj}/\lambda$). The contrast loss is even severer when the system is defocused[5], as demonstrated by the defocused $WOTF_{\text{incoh}}$ shown in Figure S1(b).

For a perfectly in-focus partially coherent system (coherence parameter $0 < s < 1$), the value of $WOTF_{\text{s}}$ is just the overlapping area of two circles with different radii and can also be represented analytically[6]:

$$WOTF_s(\bar{\mathbf{u}}) = \begin{cases} \pi s^2 & 0 \leqslant |\bar{\mathbf{u}}| \leqslant 1-s \\ s^2 \arccos\left(\frac{|\bar{\mathbf{u}}|^2+s^2-1}{2|\bar{\mathbf{u}}|s}\right) - \left(\frac{|\bar{\mathbf{u}}|^2+s^2-1}{2|\bar{\mathbf{u}}|}\right)\sqrt{s^2-\left(\frac{|\bar{\mathbf{u}}|^2+s^2-1}{2|\bar{\mathbf{u}}|}\right)^2} & \\ +s^2 \arccos\left(\frac{|\bar{\mathbf{u}}|^2-s^2+1}{2|\bar{\mathbf{u}}|s}\right) - \left(\frac{|\bar{\mathbf{u}}|^2-s^2+1}{2|\bar{\mathbf{u}}|}\right)\sqrt{1-\left(\frac{|\bar{\mathbf{u}}|^2-s^2+1}{2|\bar{\mathbf{u}}|}\right)^2} & 1-s \leqslant |\bar{\mathbf{u}}| \leqslant 1+s \end{cases} \tag{S8}$$

Figure S1(c) gives the corresponding in-focus $WOTF_s$ for different coherent parameters (which is also purely real-valued and can only image the absorption part). It can be seen that the spatial frequency cutoff of the $WOTF_s$ appears at $1+s$. Therefore, for normal brightfield imaging (for absorptive sample), it has transpired to be a good compromise when the light source image fills approximately half area of the pupil (coherent parameter $s = 0.7-0.8$) for achieving both high imaging resolution and high image contrast (especially for low spatial frequencies).

For the case of annular illumination aperture, the corresponding in-focus WOTF can be represented analytically as well. When the source is a narrow annulus with a radius of s, the WOTF is just the arc length of the ring enclosed in the objective pupil:

$$WOTF_{\text{s}}^{annular}(\bar{\mathbf{u}}) = \begin{cases} 2\pi s & 0 \leqslant |\bar{\mathbf{u}}| \leqslant 1-s \\ 2s\arccos\left(\frac{|\bar{\mathbf{u}}|^2+s^2-1}{2|\bar{\mathbf{u}}|s}\right) & 1-s \leqslant |\bar{\mathbf{u}}| \leqslant 1+s \end{cases} \tag{S9}$$

When the thickness of the annulus is $\Delta s = s$ and the outer circles of the annulus is 1, the corresponding in-focus WOTF can be calculated as the difference of $WOTF_{\text{incoh}}$ (Equation S7) and $WOTF_s$ (Equation S8):

$$WOTF_{\Delta s}^{annular}(\bar{\mathbf{u}}) = WOTF_{incoh}(\bar{\mathbf{u}}) - WOTF_s(\bar{\mathbf{u}}) =$$

$$\begin{cases} \frac{2}{\pi}\left[\arccos\left(\frac{|\bar{\mathbf{u}}|}{2}\right) - \frac{|\bar{\mathbf{u}}|}{2}\sqrt{1-\left(\frac{|\bar{\mathbf{u}}|}{2}\right)^2}\right] - \pi s^2 & 0 \leqslant |\bar{\mathbf{u}}| \leqslant 1-s \\ \frac{2}{\pi}\left[\arccos\left(\frac{|\bar{\mathbf{u}}|}{2}\right) - \frac{|\bar{\mathbf{u}}|}{2}\sqrt{1-\left(\frac{|\bar{\mathbf{u}}|}{2}\right)^2}\right] - s^2\arccos\left(\frac{|\bar{\mathbf{u}}|^2+s^2-1}{2|\bar{\mathbf{u}}|s}\right) - \left(\frac{|\bar{\mathbf{u}}|^2+s^2-1}{2|\bar{\mathbf{u}}|}\right)\sqrt{s^2-\left(\frac{|\bar{\mathbf{u}}|^2+s^2-1}{2|\bar{\mathbf{u}}|}\right)^2} & \\ +s^2\arccos\left(\frac{|\bar{\mathbf{u}}|^2-s^2+1}{2|\bar{\mathbf{u}}|s}\right) - \left(\frac{|\bar{\mathbf{u}}|^2-s^2+1}{2|\bar{\mathbf{u}}|}\right)\sqrt{1-\left(\frac{|\bar{\mathbf{u}}|^2-s^2+1}{2|\bar{\mathbf{u}}|}\right)^2} & 1-s \leqslant |\bar{\mathbf{u}}| \leqslant 1+s \\ \frac{2}{\pi}\left[\arccos\left(\frac{|\bar{\mathbf{u}}|}{2}\right) - \frac{|\bar{\mathbf{u}}|}{2}\sqrt{1-\left(\frac{|\bar{\mathbf{u}}|}{2}\right)^2}\right] & 1+s \leqslant |\bar{\mathbf{u}}| \leqslant 2 \end{cases} \quad \text{(S10)}$$

It should be noted that when the imaging system is defocused (non-paraxial), no such analytical formulas exist for those illumination settings. The corresponding $WOTFs$ can only be evaluated numerically.

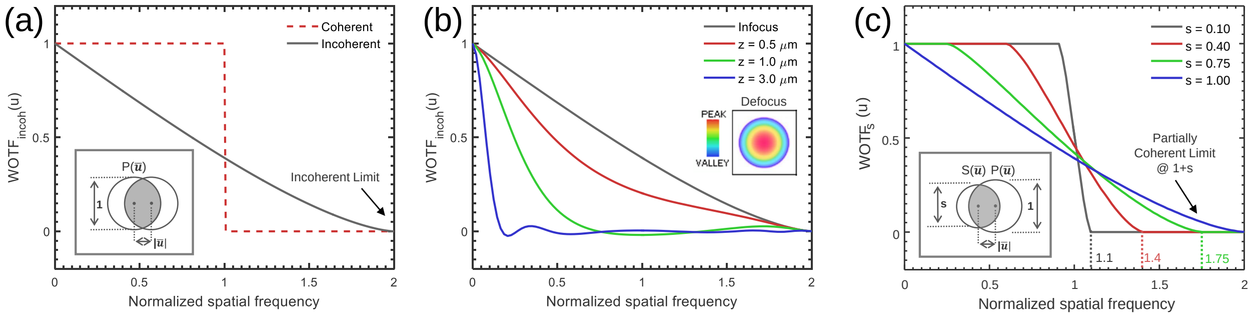

Figure S1. (a) In-focus $WOTF_{\text{incoh}}$ when the coherence parameter $s \geqslant 1$. (b) Defocused $WOTF_{\text{incoh}}$ when the coherence parameter $s \geqslant 1$. (c) In-focus $WOTF_s$ for different coherent parameters $s = 0.1$, 0.4, 0.75 and 1.0.

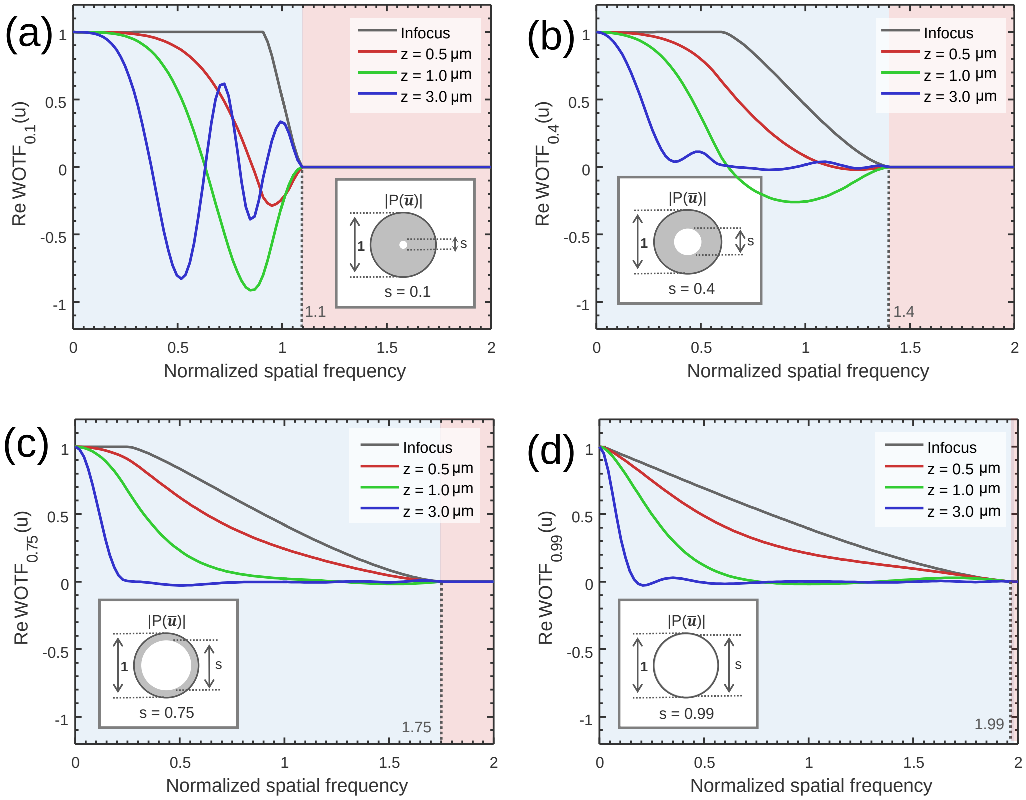

Figure S2. The real part of $WOTF_s$ (amplitude WOTF) for various coherent parameters and defocus distances ($NA_{obj} = 0.8$, $\lambda = 550nm$, the spatial frequency coordinate is normalized against the coherent resolution limit NA_{obj}/λ). (a) $s = 0.1$; (b) $s = 0.4$; (c) $s = 0.75$; (d) $s = 0.99$.

C. Comparison between Annular illumination TIE with Circular Illumination TIE in the Presence of Systematic Errors

In Figure S3, we compare the defocus images and the phase retrieval results of different illumination settings in the presence of systematic error for a small defocus distance ($\Delta z = 0.5\mu m$). The test image, imaging parameters, and the noise level are the same as in the simulation shown in Figure 8. The additional systematic error considered in this simulation includes (see top row of Figure S3): (1) Pupil aberration, which is generated from the MATLAB built-in function "peaks" with a dynamic range of $[-0.1, 0.3]$ rad; (2) Inaccurate estimation of the coherence parameter or annulus thickness (± 0.01). As can be seen from Figure S3, the model mismatch and mis-estimation about the system parameters induce obvious phase errors for the case of circular apertures. This is because that the aberration generally changes the positions of deep dips and zero-crossings in the WOTF, inducing larger phase errors due to the model mismatch during deconvolution. Furthermore, the introduction of additional measurements at large defocus distances ($3\mu m$) can no longer improve the phase reconstruction quality significantly, which is further demonstrated in Figure S4. In contrast, due to the flatness of WOTF, the systematic error has minimum impact on AI-TIE, with only a slight increase (from 0.0373 to 0.0526 for the case of $\Delta s = 0.1$) in the phase RMSE.

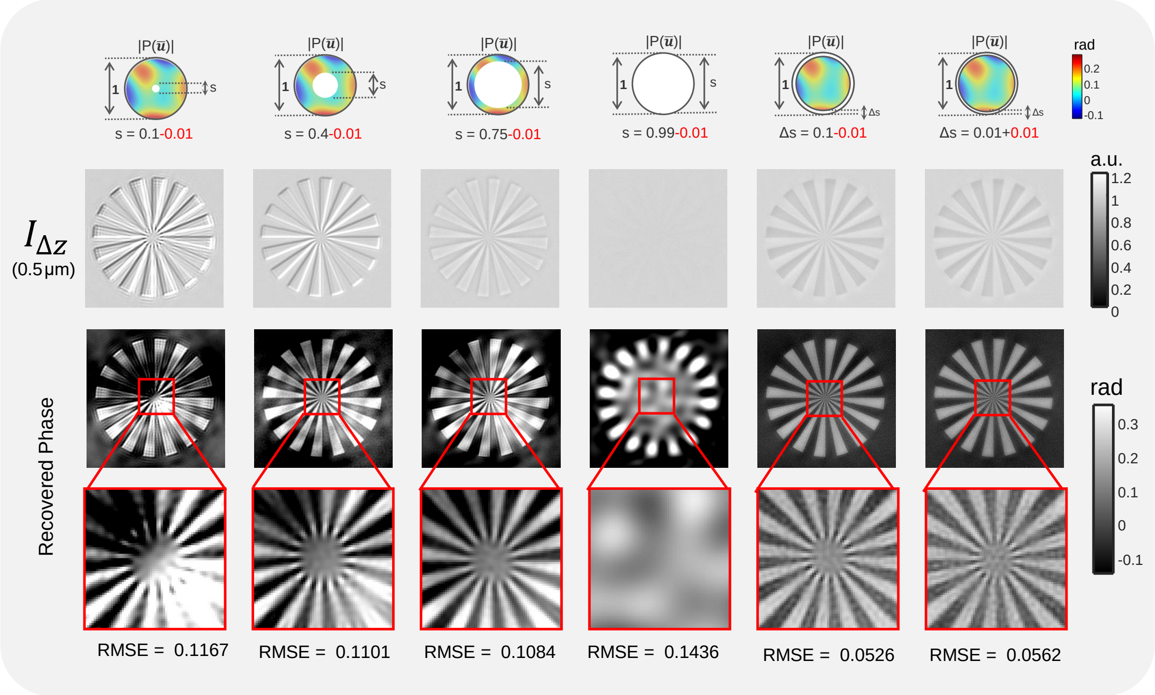

Figure S3. Comparison between annular illumination TIE with circular illumination TIE in the presence of systematic errors. The over-defocus images under different illumination settings are shown in the first row ($\Delta z = 0.5\mu m$). The reconstructed phases and the corresponding close-ups are shown in the second and third rows, respectively.

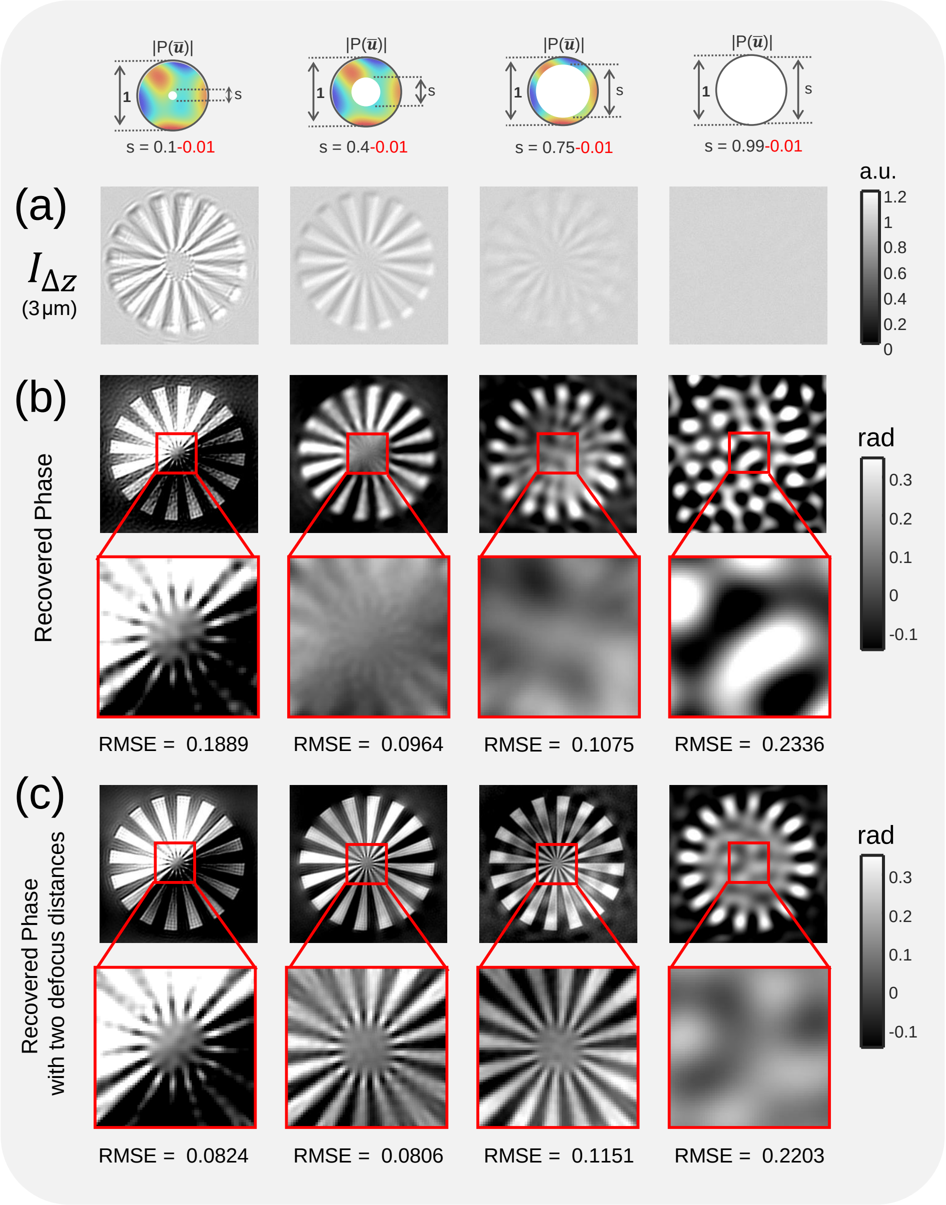

Figure S4. Comparison of over-defocus images (a) and reconstruction results of different illumination settings (b) in the presence of systematic errors when the defocus distance is large ($\Delta z = 3\mu m$). The recovered phases with both small ($\Delta z = 0.5\mu m$) and large defocus distances ($\Delta z = 3\mu m$) are shown in (c).

D. Simulation of Phase Reconstruction for Non-weak Phase object

To test the valid phase measurement range of the proposed approach, we simulate a cell sample, vary its phase from 0-1 rad to 0-10 rad, for which case the weak object approximation is violated. The system parameters are assumed to be the same as in the simulation shown in Figure 8. Figure S5 shows ideal phase maps, reconstructed phase maps with the proposed AI-TIE approach, and the comparison of the two phase profiles along the red and blue lines for the cell sample with different phase ranges. Excellent agreement between the reconstructions and ideal phase profiles can be observed when the maximum phase value is less than 5 rad. The RMSE values can be negligible compared to the true signals. When the maximum phase value increases to 10 rad, the reconstruction error becomes significant (but still acceptable, RMSE = 0.2539 rad). The simulation results suggest a valid measurement range up to 10 rad for the proposed AI-TIE, which is more than enough for imaging most of biology specimens, such as live cells and tissues.

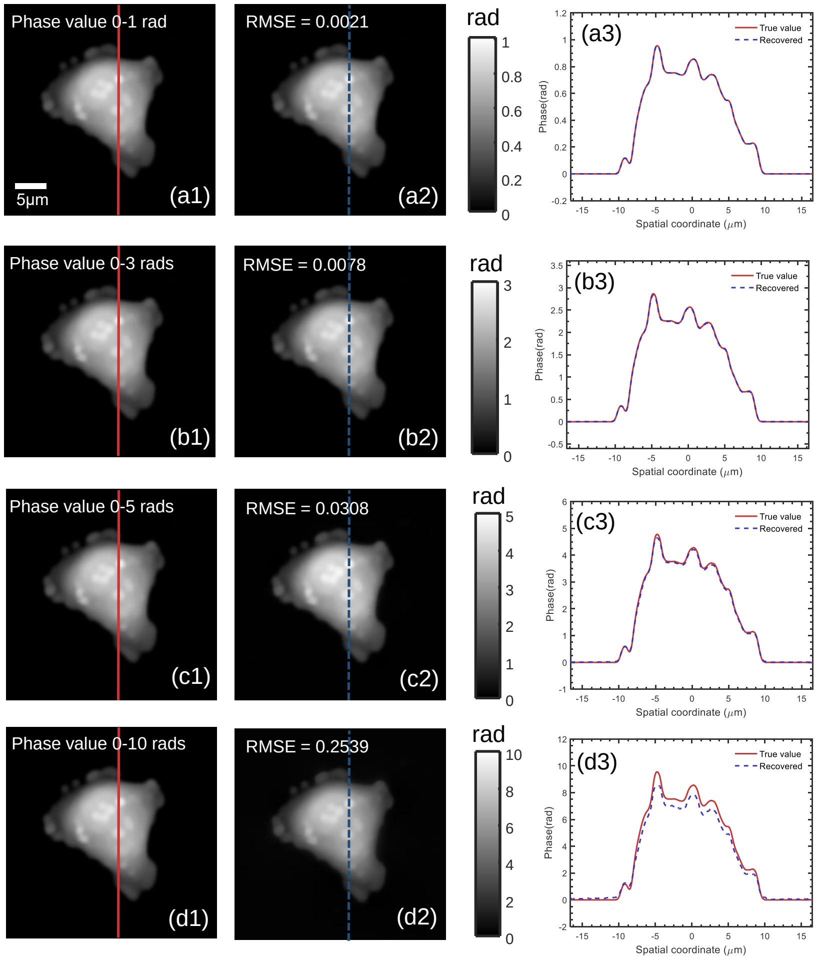

Figure S5. Simulation of the phase reconstruction for non-weak phase object. (a1)-(d1) show the ideal phase maps with different dynamic ranges (from [0,1] to [0,10]). (a2)-(d2) show the reconstructed phase maps with the proposed AI-TIE. (a3)-(d3) show the comparisons of the two phase profiles along the red and blue lines for the cell samples with different phase ranges.

E. Supplementary Videos

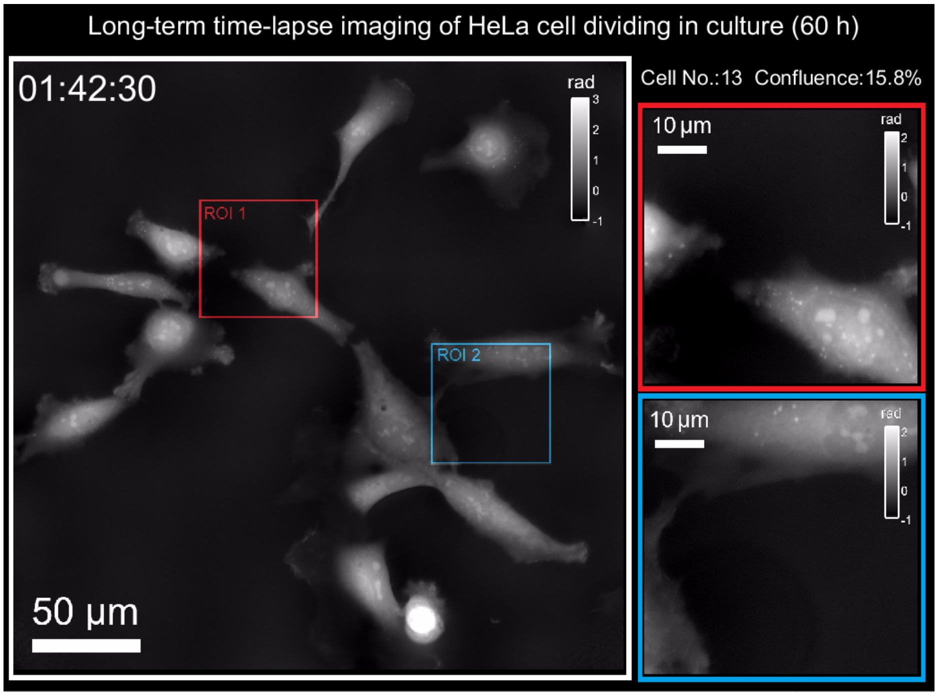

Supplementary Video 1. Time-lapse QPI video of HeLa cell dividing in culture over a 60h period at a rate of one phase image per 30 second. Two regions of interests (ROI 1 and ROI 2) are enlarged and shown on the right (see also Figure 14).

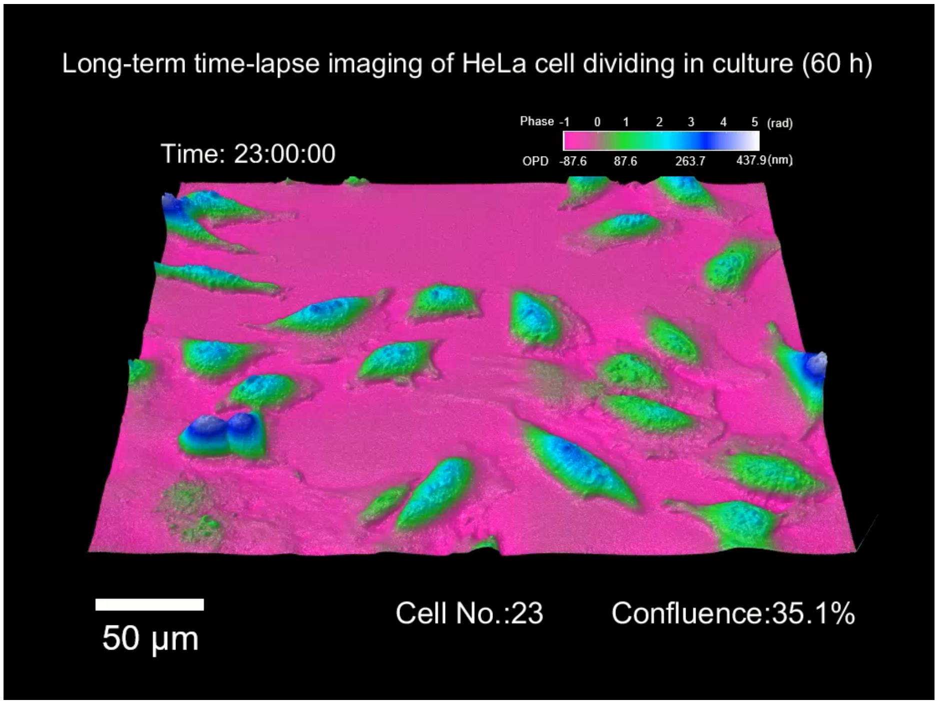

Supplementary Video 2. 60-hour time-lapse 3D movie of HeLa cell dividing in culture. The pseudo-color 3D rendering represents phase (optical path length) profiles of cells.

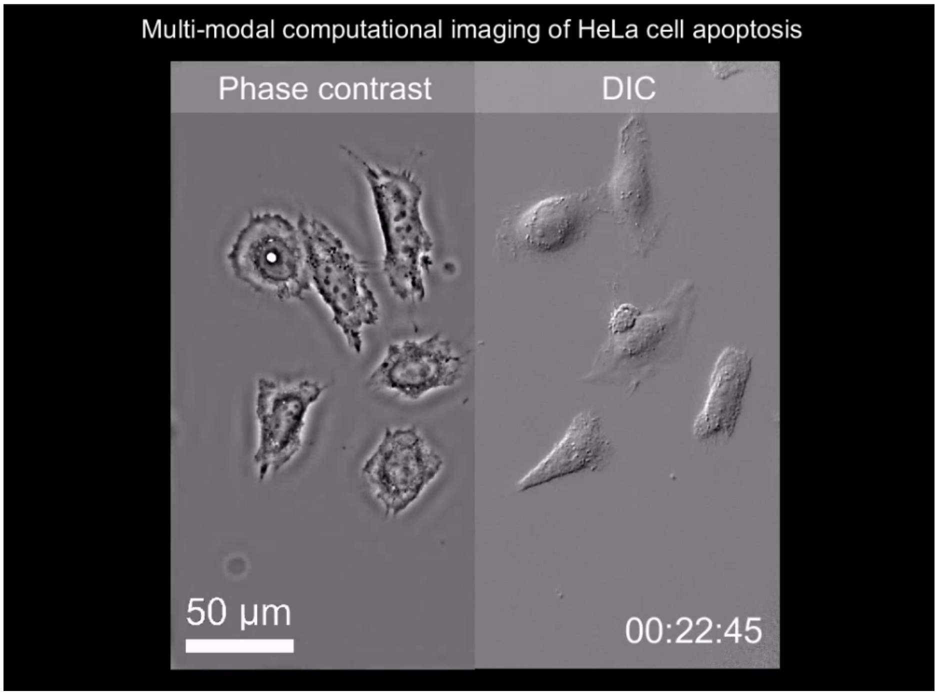

Supplementary Video 3. Multi-modal (quantitative phase, phase contrast, and DIC) computational imaging of apoptotic HeLa cells induced by paclitaxel (see also Figure 15). The phase is reconstructed every second over one hour period (1 frame/s).

References

1. Mandel, L. & Wolf, E. *Optical Coherence and Quantum Optics* (Cambridge University Press, 1995).

2. Goodman, J. W. *Statistical optics* (John Wiley and Sons, 2015).

3. Zuo, C., Chen, Q., Tian, L., Waller, L. & Asundi, A. Transport of intensity phase retrieval and computational imaging for partially coherent fields: the phase space perspective. *Opt. Lasers in Eng.* **71**, 20–32 (2015).

4. Hopkins, H. On the diffraction theory of optical images. In *Proc. R. Soc. A*, vol. 217, 408–432 (The Royal Society, 1953).

5. Hopkins, H. The frequency response of a defocused optical system. In *Proc. R. Soc. A*, vol. 231, 91–103 (The Royal Society, 1955).

6. Sheppard, C. J. Defocused transfer function for a partially coherent microscope and application to phase retrieval. *JOSA A* **21**, 828–831 (2004).
